# Supplementary material for: Enhancing the scalability of distance-based link prediction algorithms in recommender systems through similarity selection
Source: PLoS One. 2022 Jul 28;17(7):e0271891. doi: 10.1371/journal.pone.0271891 (PMC9333256; doi:10.1371/journal.pone.0271891)
Supplement: Attachment — Submitted filename: response-to-reviwers_v2.pdf [file pone.0271891.s002.pdf]

Original Manuscript ID: PONE-D-21-27341

Original Article Title: "Link prediction in recommender systems based on user-rating distance and similarity selection"

To: Editors

Re: Response to reviewers

Dear Editor,

Thank you for allowing a resubmission of our manuscript, with an opportunity to address the reviewers' comments.

We are uploading (a) our point-by-point response to the comments (below) (response to reviewers), (b) an updated manuscript with yellow highlighting indicating changes.

More importantly, the authors would like to thank the anonymous reviewers for their valuable remarks and comments, which significantly contributed to the quality of the paper. We consider the anonymous reviewers as anonymous coauthors of our work.

Best regards,

Zhan Su, Zhong Huang, Jun Ai et al.

Reviewer#1, Concern # 1: Page 2, line 46, what does it means by "reducing the sparsity of the Slope One method."

Author response: Thank you for the comment. The main problem addressed in this paper is the scalability (the number of used neighbors in prediction) and prediction accuracy of model-based collaborative filtering. We have further elaborated on this goal by modifying the abstract and contribution sections.

Author action: We have deleted the inaccurate expressions in the revised paper.

Reviewer#1, Concern # 2: The authors mentioned some technical problems, but feel a little mixed, if authors can highlight two or three technical difficulties, and in the performance evaluation targeted to show the advantages of the proposed method, it would be better.

Author response: Thank you for the comment. The main technical problem addressed in this paper is the scalability (the number of used neighbors in prediction) and prediction accuracy of model-based collaborative filtering. We have further elaborated on this goal by modifying the abstract and contribution sections.

Author action: In accordance with the reviewers' comments, we have revised the relevant expressions and marked them in the revised manuscript. On Page 3, marked yellow.

Reviewer#1, Concern # 3: The main contribution of this article is mentioned on page 3, but the contribution should be linked to the requirements, as well as the problems that these contributions can answer.

Author response:

Thank you for the comment.

Author action: As the reviewer suggested, we have revised the contribution and marked them in the revised manuscript. On Page 3, marked yellow.

Reviewer#1, Concern # 4: Have the authors tried to change the similarity-selection equation (3) to other forms? For example, use |s(u,v)|>=T_h instead of s(u,v). Negative correlation is likely to be helpful in prediction.

Author response:

Thank you for the comment. We considered several similar techniques of filtering during the experimental exploratory phase, but the results were not promising. The findings of a typical comparative experiment are shown in the following figures (MAE and NDCG, the method reviewer suggested labeled as DSS-abs, the one we use in the paper is labeled as DSS), which illustrates that filtering neighbors with large absolute values retains both positively and negatively correlated neighbors (|s(u,v)|>=T_h), but will cause the prediction error to become larger and NDCG ranking is little worse than the original Slope One. Thus, we did not include other forms of experimental results in the revised manuscript.

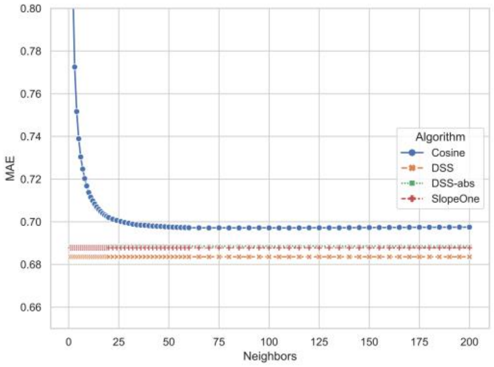

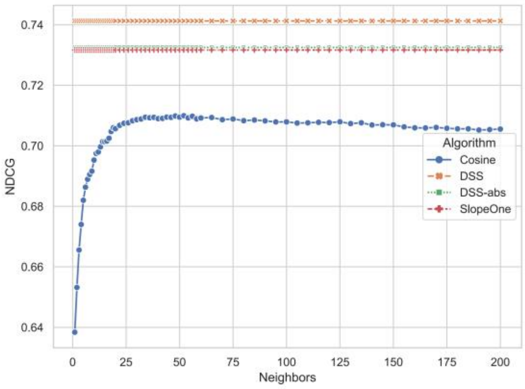

Author action: None

Reviewer#1, Concern # 5: Authors need to add more discussion on why the similarity selection improves the overall performance.

Author response:

Thank you for the comment.

Author action:

As suggested by the reviewer, we've added a corresponding discussion and analysis in the conclusion section. On Page 15 and 16, marked yellow.

Reviewer#2, Concern # 1: Refer to Algorithm 1, please provide more discussion on top-k prediction in recommendation list, in your work. Please give more explanation on it in the experimental results.

Author response:

Thank you for the comment.

Author action:

As suggested by the reviewer, we have added discussion of the Top-k prediction in the revised paper. On Page 9, marked yellow.

Reviewer#2, Concern # 2: Could you please add AUC of the results in the experiments.
Author response:

Thank you for the advice.

Author action:

As suggested by the reviewer, we've added Recall, Precision, Accuracy and ROC/AUC in the revised paper. On Page 12,13 and 14, add Fig 8, 9, 10 and 11, marked yellow.

Reviewer#2, Concern # 3: Please discuss in more detail the information you have employed in your work. As you know, there are some information in MovieLens. Have you only used rating rate?

Author response:

Thank you for the comment. We use only the ratings data from the MovieLens data set.

Author action:

We have added discussion in the revised paper to address all the detail of experiments. We also increased the number of users used in the experiments, and redrew completely new figures of the experiments. On Page 7, marked yellow.

Reviewer#2, Concern # 4: Does the method help to solve the sparsity problem? Furthermore, there is a long tailed distribution for number of ratings. How does the proposed method deal with ling tailed ratings?

Author response:

Thank you for the advice.

Our main concern addressed in this paper is the scalability of model-based collaborative filtering, the proposed method aims to reduce the number of required neighbors of prediction, as well as improving the prediction accuracy. Thus, the method doesn't help with the sparsity problem.

Additionally, we don't currently consider the long-tail distribution of scores, but we'd would like to look into it in the future.

Author action:

We've included the suggestion in the conclusion section, along with a forecast for future study, and we're grateful to the reviewers for his or her insightful advice. On Page 16, marked yellow.

Reviewer#2, Concern # 5: It is more interesting to see the pitfalls of the method and future line of this study in Conclusion section.

Author response:

Thank you for the advice. The drawback of the algorithm is that the computational steps are added, but the reduction of the prediction error is not significant, and the similarity in the distance-based prediction process is not utilized in the previous calculation.

Author action: Based on the reviewers' suggestions, we have added a relevant discussion in the conclusion section. On Page 16, marked yellow.
