# Supplementary material for: Enhancing the scalability of distance-based link prediction algorithms in recommender systems through similarity selection
Source: PLoS One. 2022 Jul 28;17(7):e0271891. doi: 10.1371/journal.pone.0271891 (PMC9333256; doi:10.1371/journal.pone.0271891)
Supplement: Attachment — Submitted filename: response-to-reviwers_v2.pdf [file pone.0271891.s003.pdf]

Original Manuscript ID: PONE-D-21-27341R1

Original Article Title: "Enhancing the scalability of distance-based link prediction algorithms in recommender systems through similarity selection"

To: Editors

Re: Response to reviewers

Dear Editor,

Thank you for allowing a resubmission of our manuscript, with an opportunity to address the reviewers' comments.

We are uploading (a) our point-by-point response to the comments (below) (response to reviewers), (b) an updated manuscript with yellow highlighting indicating changes, (c) a clean revised manuscript.

More importantly, the authors would like to thank the anonymous reviewers for their valuable remarks and comments, which significantly contributed to the quality of the paper. We consider the anonymous reviewers as anonymous coauthors of our work.

Best regards,

Zhan Su, Zhong Huang, Jun Ai et al.

Reviewer#1, Comment# 1: The authors have revised this manuscript carefully and stated how they revised in detail as the reviewers suggested, and I am satisfied with this revision in principle. The manuscript also includes a few writing mistakes, and I suggest the authors carefully to proofreading this manuscript.

Author response: Thank you for the comment. We have carefully proofread the article.

Author action: Writing errors and typos have been corrected in the revised paper, which were marked yellow in the marked manuscript.

Reviewer#1, Comment # 2: Line 67 on Page 10: The statement of "Not only is the Slope One method simple to implement, but it is also highly effective" is suggested to be revised to "The Slope One method is not only simple to implement, but also highly effective".

Author response: Thank you for the comment.

Author action: The issues pointed out by the reviewers have been corrected in the revised manuscript.

Reviewer#1, Concern # 3: The citations of figures in this manuscript are inconsistent, such as "Fig 2", "Figure 3", "figure 3", etc.

Author response: Thank you for the comment.

Author action: The issues pointed out by the reviewers have been corrected in the revised manuscript.

Reviewer#1, Concern # 4: The metrics, such as NDCG (Line 290 on Page 18), DCG (Line 295 on Page 18), should be italic.

Author response: Thank you for the comment.

Author action: The issues pointed out by the reviewers have been corrected in the revised manuscript.

Reviewer#2,

No further comments
